# Author Correction: Mid-infrared supermirrors with finesse exceeding 400 000

**DOI:** 10.1038/s41467-024-54245-7

**Published:** 2024-11-29

**Authors:** Gar-Wing Truong, Lukas W. Perner, D. Michelle Bailey, Georg Winkler, Seth B. Cataño-Lopez, Valentin J. Wittwer, Thomas Südmeyer, Catherine Nguyen, David Follman, Adam J. Fleisher, Oliver H. Heckl, Garrett D. Cole

**Affiliations:** 1 Thorlabs Crystalline Solutions, 114 E Haley St., Suite G, Santa Barbara, CA 93101 USA; 2 Christian Doppler Laboratory for Mid-IR Spectroscopy and Semiconductor Optics, Faculty Center for Nano Structure Research, Faculty of Physics, University of Vienna, Boltzmanngasse 5, A-1090 Vienna, Austria; 3 Vienna Doctoral School in Physics, University of Vienna, Boltzmanngasse 5, A-1090 Vienna, Austria; 4 National Institute of Standards and Technology, 100 Bureau Drive, Gaithersburg, MD 20899 USA; 5 Laboratoire Temps-Fréquence, Institut de Physique, Université de Neuchâtel, Avenue de Bellevaux 51, 2000 Neuchâtel, Switzerland

**Keywords:** Optical materials and structures, Materials for optics, Infrared spectroscopy

Correction to: *Nature Communications* 10.1038/s41467-023-43367-z, published online 06 December 2023

The original version of the published Article had incorrect assignment of references in Table 1 (see below)

**Table 1:** Performance of comparable cavity ring-down spectrometers operating near 4.5 μm

Reference	*α*_0_ at 1 s (cm^−1^ Hz^−1/2^)	*L* (m)	*α*_0_ at 1 s for *L* = 10 cm (cm^−1^ Hz^−1/2^)
INO-CNR^18^	5.0 × 10^−9^	1	5.0 × 10^-7^
VTT^19^	2.1 × 10^−9^	0.4	3.4 × 10^-8^
Nagoya^20^	1.1 × 10^−8^	0.11	1.3 × 10^-8^
NIST^21^	2.6 × 10^−11^	1.5	5.9 × 10^-9^
LLNL^15,22^	1.2 × 10^−10^	0.67	5.3 × 10^-9^
This work	6.0 × 10^−11^	0.79	3.7 × 10^-9^

Here the absorption coefficient at 1 s integration, cavity length, and normalized performance for each system are shown. The ultralow optical loss of our crystalline coatings yields the lowest normalized noise-equivalent absorption.

For reading Table 1 correctly, the following list of reference changes are applicable

- In Line 1, INO-CNR – reference changes from 18 to 20
- Line 2, VTT – reference changes from 19 to 21
- Line 3, Nagoya – reference changes from 20 to 22
- Line 4, NIST – reference changes from 21 to 23
- Line 5, LLNL – reference changes from 15&22 to 17&24, respectively.

The original article has been corrected.

