# Supplementary material for: Mid-infrared supermirrors with finesse exceeding 400 000
Source: Nat Commun. 2023 Dec 6;14:7846. doi: 10.1038/s41467-023-43367-z (PMC10700499; doi:10.1038/s41467-023-43367-z)
Supplement: Supplementary file 1 — Supplementary Information [file 41467_2023_43367_MOESM1_ESM.pdf]

SUPPLEMENTAL INFORMATION FOR:

Mid-infrared supermirrors with finesse exceeding 400 000

G.-W. Truong et. al.

Estimation of mirror transmittance

As described in the Methods section, we estimate mirror transmittance *T* based on a best-fit transmission matrix model (TMM) seeded with measurements of both a broadband transmittance spectrum via Fourier-transform spectrometer (FTS) and Bragg-mirror layer thicknesses via scanning-electron microscope (SEM). Suppl. Fig. 1(a) shows the data and best-fit TMM model in a broader spectral range, while Suppl. Fig. 1(b) illustrates how we overcome SNR-limitations in FTS transmittance measurements using our approach.

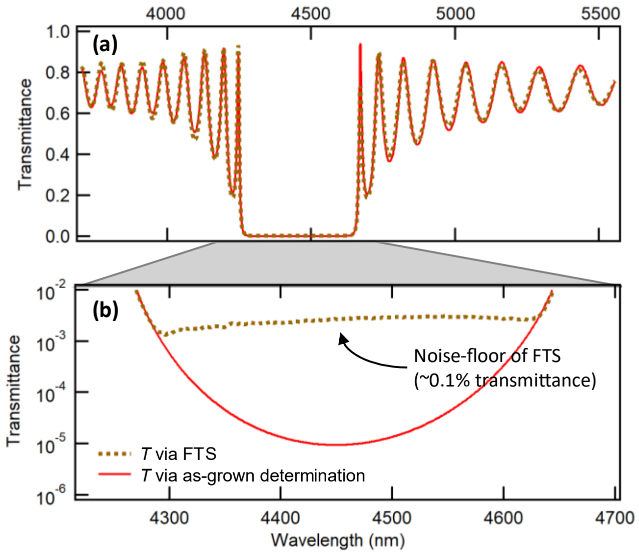

Suppl. Fig. 1. (a) Broadband FTS transmittance of HR1, one of the all-crystalline supermirrors, compared to a TMM computation pinned to SEM, and FTS measurements, as well as known refractive indices. (b) Zoomed-in view near the transmittance minimum, showing that the FTS lacks sufficient transmittance accuracy within the stop band.

Time-domain ringdown data

Suppl. Fig. 2(a-h) show typical time-domain optical decay signals, fit residuals, and the distribution of measured time constants for measurements using our lossmeter (cavity length of 145 mm), which was used to determine mirror performance. Suppl. Fig. 2(i-j) shows a typical ring-down curve and fit residuals for the ring-down spectrometer (cavity length of 79 cm), which was used for our proof-of-principle spectroscopy measurements.

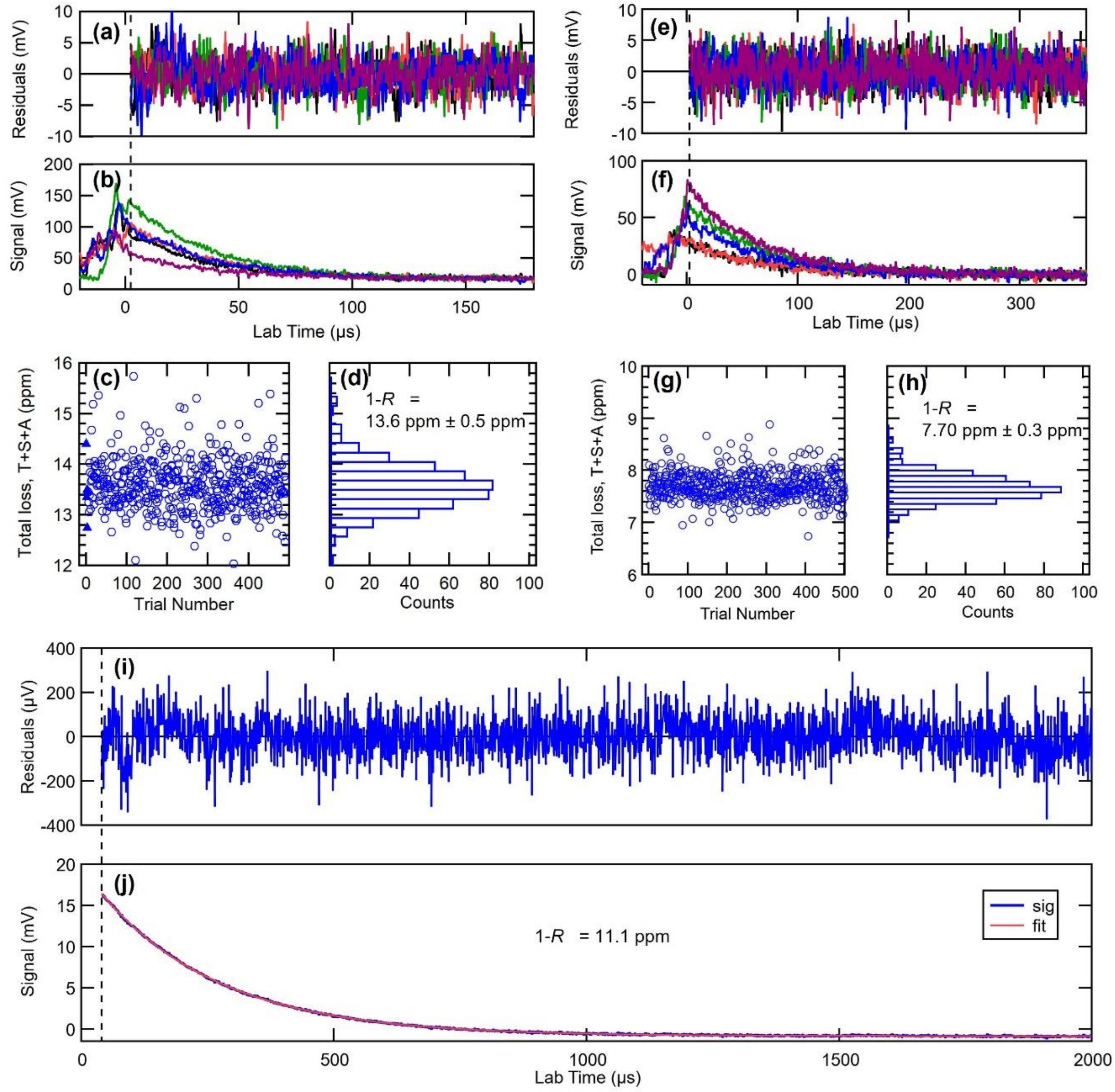

Suppl. Fig. 2. (a-d): Consecutive ring-downs taken at a particular location on the all-crystalline mirrors. SNR ~ 40, depending on trace. (e-h): Consecutive ring-downs taken at a particular location on the hybrid mirrors. SNR ~ 20. (i-j): a single ring-down measurement taken at a particular location on the hybrid mirrors using the evacuated 79-cm cavity spectrometer. The small difference in the measured reflectivity of the hybrid mirrors taken from the 145-mm coating lossmeter and in the 79-cm spectrometer is due to difference in experimental conditions (such as spot size and location). All ring-downs were taken at λ = 4.45 µm.
